# An Empirical Orthogonal Function-Based Algorithm for Estimating Terrestrial Latent Heat Flux from Eddy Covariance, Meteorological and Satellite Observations

**DOI:** 10.1371/journal.pone.0160150

**Published:** 2016-07-29

**Authors:** Fei Feng, Xianglan Li, Yunjun Yao, Shunlin Liang, Jiquan Chen, Xiang Zhao, Kun Jia, Krisztina Pintér, J. Harry McCaughey

**Affiliations:** 1 State Key Laboratory of Remote Sensing Science, College of Global Change and Earth System Science, Beijing Normal University, Beijing, 100875, China; 2 State Key Laboratory of Remote Sensing Science, School of Geography, Beijing Normal University, Beijing, 100875, China; 3 Department of Geographical Sciences, University of Maryland, College Park, MD, 20742, United States of America; 4 Landscape Ecology & Ecosystem Science (LEES) Lab, Center for Global Change and Earth Observations (CGCEO), Michigan State University, East Lansing, MI, 48823, United States of America; 5 Institute of Botany and Ecophysiology, Szent István University, 2100 Páter K.u.1., Gödöllő, Hungary; 6 Department of Geography, Queen’s University, Mackintosh-Corry Hall, Room E112, Kingston, Ontario, Canada; 7 MTA-SZIE Plant Ecology Research Group, 2103, Gödöllő, Hungary; University of Aveiro, PORTUGAL

## Abstract

Accurate estimation of latent heat flux (LE) based on remote sensing data is critical in characterizing terrestrial ecosystems and modeling land surface processes. Many LE products were released during the past few decades, but their quality might not meet the requirements in terms of data consistency and estimation accuracy. Merging multiple algorithms could be an effective way to improve the quality of existing LE products. In this paper, we present a data integration method based on modified empirical orthogonal function (EOF) analysis to integrate the Moderate Resolution Imaging Spectroradiometer (MODIS) LE product (MOD16) and the Priestley-Taylor LE algorithm of Jet Propulsion Laboratory (PT-JPL) estimate. Twenty-two eddy covariance (EC) sites with LE observation were chosen to evaluate our algorithm, showing that the proposed EOF fusion method was capable of integrating the two satellite data sets with improved consistency and reduced uncertainties. Further efforts were needed to evaluate and improve the proposed algorithm at larger spatial scales and time periods, and over different land cover types.

## 1. Introduction

Terrestrial latent heat flux (LE), the flux of heat from the Earth’s surface to the atmosphere that is associated with soil evaporation and plant transpiration, and is a key component of the hydrological and carbon cycles [1, 2]. Accurate and temporally continuous estimation of LE is critical for understanding the interactions between the land surface and the atmosphere and improving water use efficiency [3, 4].

Many LE products were developed at various temporal and spatial resolutions during the past several decades [5–11], which were needed to study long-term regional and global climate change [12]. Extensive evaluations of these products were conducted [13–20]. Chen et al. [4] compared eight evapotranspiration (ET) models (equivalent to LE) and found significant inconsistencies among the models, largely due to the driving factors. Long et al. [14] assessed the uncertainty in LE estimates from four land surface models, including two remote sensing-based products (MODIS and advanced very high resolution radiometer (AVHRR)), and Gravity Recovery and Climate Experiment (GRACE), by inferring ET from water budget, and found that uncertainty of remote sensing products was approximately 10–15 mm/month. Furthermore, spatial LE interactions were often ignored in most satellite-based LE models [21]. Ershadi et al. [21] used the surface energy balance system (SEBS) and Landsat images to investigate the effects of aggregation from fine (<100 m) to medium (~1 km) scales. From several common spatial interpolation algorithms, the simple average method preserved most accurately the spatial pattern of LE compared to the nearest neighbors and bilinear or bicubic interpolation methods.

Efforts were made to improve the quality of LE products by developing advanced LE retrieval algorithms [6, 9–11] and using data assimilation methods [22–29]. Data assimilation involved numerical models that incorporate measured data to produce final results for forecasting or analysis [30]. Caparrini et al. [23] used data assimilation to obtain LE, sensible heat and ground heat flux. Similar studies on surface temperature, sensible heat flux, and LE were also performed [25–27]. However, integrating advantages from existing LE products to improve data accuracy and integrity was the main goal. Previous studies showed that averaged LE was more accurate than individual LE models [20, 24]. Cammalleri et al. [22] combined multi-platform remote sensing thermal infrared data to estimate daily field-scale LE data using a spatial and temporal adaptive reflectance fusion model (STARFM). Yao et al. [28] combined five process-based LE algorithms using Bayesian averaging method. However, these methods usually failed to account for spatial and temporal correlations of LE when integrating satellite LE products.

In geosciences, empirical orthogonal function (EOF) method deals with both, temporal and spatial patterns [31, 32]. EOF was first used in meteorology to decompose a space-time field into spatial patterns and associated time indices. Incorporating both spatial and temporal correlations, Chen et al. [33] developed an extended EOF that became a powerful tool to extract dynamic structure, including trends, oscillations, propagating structures and to filter data. Smith et al. [34] used EOF analysis to solve the problem of missing data. Beckers and Rixen [35] developed a “self-consistent” and “parameter-free” EOF interpolation method, data interpolating EOF (DINEOF), which had proven useful for oceanographic data analysis [36]. Wang et al. [37] used hierarchical EOFs (HEOFs) to integrate LAI from MODIS and Carbon cycle and Change in Land Observational Products from an Ensemble of Satellites (CYCLOPES) to improve the quality of satellite based LAI data, which resulted in increase of R^2^ (from 0.75 to 0.81) and in decrease of root mean square error (rmse) from 1.04 to 0.71.

In this paper, we propose an EOF-based data-fusion method that combines the major spatial and temporal patterns of different LE data to generate a consistent and high accuracy dataset. Errors in satellite based LE products might arise from the use of different driving factors or empirical coefficients. Therefore two process-based LE algorithms were selected to perform the data fusion: the MOD16 algorithm based on the PM approach [38] and the PT-JPL algorithm based on the PT approach [39]. The objectives of this study were to (1) compare the MOD16 and PT-JPL algorithms at FLUXNET sites; (2) evaluate the performance of the proposed EOF fusion method by comparing it to MOD16, PT-JPL and a simple fusion method; and (3) assess the limitations of proposed method.

## 2. Materials and Methods

### 2.1 The principle of the empirical orthogonal function (EOF)

A fundamental advantage of the EOF-based method was to reconstruct the original data by minimizing the noise and the gaps. EOF incorporated principal component analysis (PCA), but also considered the temporal and spatial characteristics of the data [31]. LE data was stored in a *P × N* matrix (*A*), where *s* ∈ [1, P] and represents space and *t* ∈ [1, N] denotes time. Matrix A was decomposed by singular value decomposition, which is a commonly used method in linear algebra:

$$A=ZSH^{T}$$ (1)

where *Z* stands for the left singular vectors (EOFs). *S* for a diagonal matrix containing the singular values sorted in descending order, and *H* for the right singular vectors (PCs). EOFs represents the spatial domain, whereas PCs represents the temporal domain. Thus, the spatial and temporal components were separated. Singular-value decomposition was also used to filter noise.

We expanded the EOF to emphasize temporal information [33] by subsetting A in time windows (W) and combining the subsets in a new matrix.

$$\left(a_{1}\;a_{2}\ldots a_{N}\right)$$ (2)

denotes time series of LE at a specific location, from which a matrix was built using a window length of W,

$$A=\left(\begin{array}{ccc} \begin{array}{c} a_{1}\\ a_{2} \end{array} & \begin{array}{c} \begin{array}{cc} a_{2} & \cdots \end{array}\\ \begin{array}{cc} a_{3} & \ldots \end{array} \end{array} & \begin{array}{c} a_{N-W+1}\\ a_{N-W+2} \end{array}\\ \vdots & \begin{array}{cc} \vdots & \ddots \end{array} & \vdots \\ a_{W} & \begin{array}{cc} a_{W+1} & \ldots \end{array} & a_{N} \end{array}\right)$$ (3)

We applied this process to all spatial points, obtaining matrix *A*′ with dimension *W × (N −W + 1)*. In this study, the EOF analysis was conducted with this matrix *A*′.

$$A\prime =\left(\begin{array}{ccc} \begin{array}{cc} \begin{array}{c} A_{1,1}\\ A_{1,2}\\ \vdots \end{array} & \begin{array}{c} A_{1,2}\\ A_{1,3}\\ \vdots \end{array} \end{array} & \begin{array}{c} \ldots \\ \ldots \\ \ddots \end{array} & \begin{array}{c} A_{1,N-W+1}\\ A_{1,N-W+2}\\ \vdots \end{array}\\ \begin{array}{cc} \begin{array}{c} A_{1,W}\\ A_{2,1}\\ \vdots \end{array} & \begin{array}{c} A_{1,W+1}\\ A_{2,2}\\ \vdots \end{array} \end{array} & \begin{array}{c} \ldots \\ \ldots \\ \ddots \end{array} & \begin{array}{c} A_{1,N}\\ A_{2,N-W+1}\\ \vdots \end{array}\\ \begin{array}{cc} A_{P,W} & A_{P,W+1} \end{array} & \ldots & A_{P,N} \end{array}\right)$$ (4)

Traditional EOF analysis addresses matrices that contain no missing data. However, remote sensing data often does not satisfy this requirement, thus a modified EOF analysis (DINEOF) [35] was employed which uses iterative algorithms to estimate missing data. Before iteration, missing data were replaced with zeroes. Then, the following iterative algorithm was applied to the input matrix data with mean subtraction,

$${\left(X_{a}\right)}_{ij}={\left(ZS_{N}H_{N}^{T}\right)}_{ij}=\sum_{k=1}^{N}p_{k}{\left(Z_{k}\right)}_{i}{\left(H_{k}^{T}\right)}_{j},\left(i,j\right)\in I$$ (5)

where *i* and *j* are the location and time of the missing data, respectively; (*X*_*a*_)_*ij*_ is the reconstructed data using the leading components (*N*) of the data; and *P* is the eigenvalue.

Traditional EOF analysis usually employes matrices that contain few spatial points, i.e., the images had coarse spatial resolution. When handling remote sensing data, the number of spatial point increases and the computational time becomes challenging. A modified HEOF was used to solve this problem. HEOFs [37] worked on two levels: coarse and fine-resolution. However, coarse resolution data also required considerable memory capacity. We simplified the original HEOF procedure by dividing the dataset into small subsets and applying an EOF to each of them. Because other subset information could not be used, we used the relative information.

### 2.2 The Framework of EOF fusion

Implementation of the EOF-based algorithm requires the following steps: (1) Forming the necessary matrix for EOF analysis from a time series of the satellite data (*A*). One year’s data of MOD16 and PT-JPL was randomly selected. (2) Defining the number of leading components of each LE algorithm and the window length. (3) Intergration of MOD16 and PT-JPL output matrixes by their principal components.

**Fig 1:**
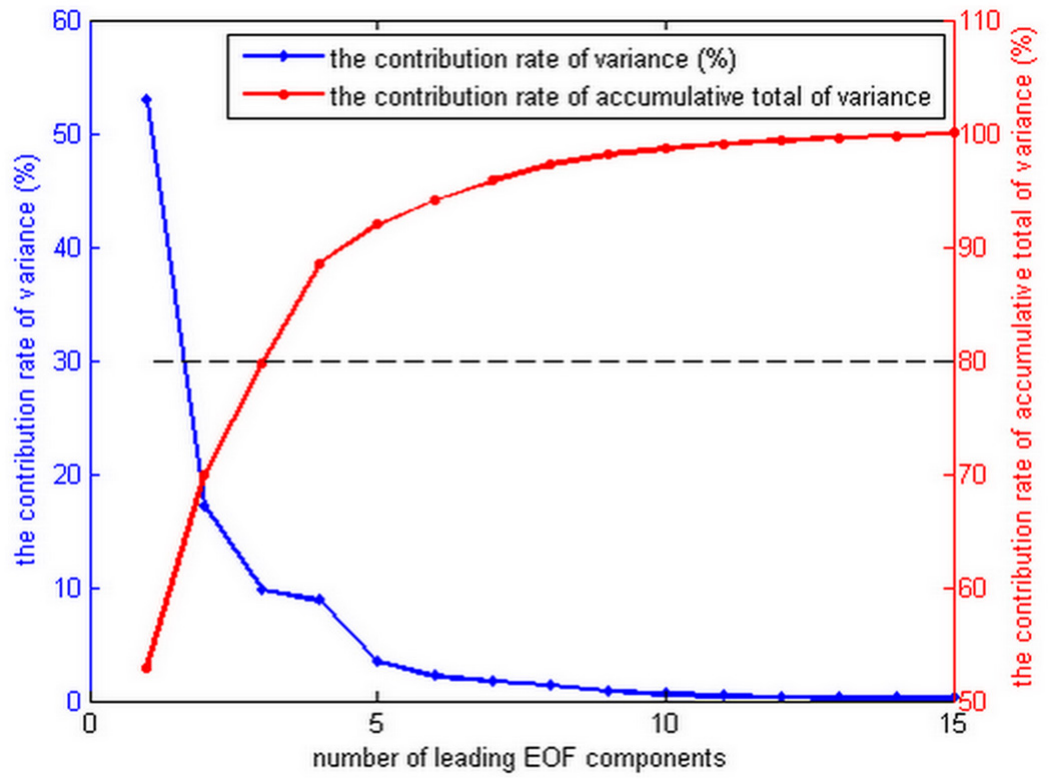
Coefficient of determination of latent heat fluxes (PT-JPL model) as a function of the number of leading EOF components. The left Y-axis is the contribution rate of covariance for each single EOF components (blue). The right Y-axis is the contribution rate of cumulative total of variance (red).

We selected one year (2005) of data for MOD16 and PT-JPL, to test the proposed EOF method. The good overall performance of PT-JPL model was reported previously [19, 40, 41]. In the proposed EOF method 80% of the PT-JPL components and 20% of the MOD16 components were used. From PT-JPL the three leading components explaining about 80% of the total variance were selected (Fig 1) [42]. In the case of MOD16, leading components were selected from the same position as in PT-JPL, which meant twelve leading components that explain about 20% of the total variance [42]. Low LE values of PT-JPL during spring and winter caused negative LE values during EOF reconstruction process, thus these values were replaced by the main patterns of MOD16. Choosing *W = 1* means that no temporal information is used and the expanded EOF method corresponds to the simple EOF method. To both reduce the computation time and emphasize temporal information the window size was set to 4. The EOF algorithm then was applied to a 200km × 200 km region for an entire year. For the purpose of validation, we included the domain around the FLUXNET sites, where each subset contained one FLUXNET site. We used a simple averaging (SA) model to integrate MOD16 and PT-JPL. The SA method was a simple fusion algorithm taking a constant weight (0.5) for each model. (S1 File)

## 3. Data and Analysis

### 3.1 Satellite Data

Satellite LE products included MODIS with 1 km spatial resolution, University of California Berkeley (UCB) with 0.5 degree spatial resolution, Global Land Evaporation: the Amsterdam Methodology (GLEAM) with 0.5 degree spatial resolution, Atmospheric water balance (AWB) with 2.5 degree spatial resolution, and University of Maryland (MAUNI) with 1 degree spatial resolution. The reanalysis products include the Global Modeling and Assimilation Office's (GMAO)- modern-era retrospective analysis for research and applications (MERRA) with ½ degree latitude × ⅔ degree longitude spatial resolution, interim ERA- the latest global atmospheric reanalysis produced by (ECMWF) with approximately 80 km degree spatial resolution, The National Centers for Atmospheric Prediction/National Center for Atmospheric Research (NCAR/NCEP) with 2.5 degree spatial resolution, MERRA-Land Reanalysis (M-LAND) with ½ degree latitude × ⅔ degree longitude spatial resolution. However, most global LE products had high uncertainties [2] and low resolution. Satellite data was used to estimate land surface variables, which were used as inputs for LE algorithm. Satellite data based LE algorithms were easy to operate for routine, long-term mapping of LE with different spatial scales. However, models structure and physical parameterizations of LE algorithms influenced the accuracy of these products. Dirmeyer et al., 2013 [43] found that model parameterizations in Penman-Monteith equation based LE algorithms influenced the accuracy assessment of LE.

The MODIS LE product algorithm (MOD16) was based on a beta version [5] developed from Cleugh et al. [44] using the PM model [38]. Mu et al. (2011) [11] improved the beta version by: (1) simplifying the calculation of the vegetation cover fraction with FPAR; (2) calculating LE as the sum of daytime and nighttime components; (3) improving calculations of aerodynamic, boundary-layer, and canopy resistance; (4) estimating the soil heat flux using available energy and simplified NDVI; (5) dividing the canopy into wet and dry components; (6) separating moist soil surfaces from saturated wet ones. The MOD16 algorithm was successfully extended to generate MODIS global terrestrial LE product from MODIS land cover, albedo, LAI/FPAR, and a GMAO daily meteorological reanalysis data set [11].

To avoid the complexity of parameterizing aerodynamic and surface resistance, Priestley and Taylor [45] reduced the atmospheric control term in the PM equation and added an empirical factor to design a simple LE algorithm. Based on this algorithm, Fisher et al. [6] proposed a novel PT-based LE algorithm (Priestley-Taylor LE algorithm of Jet Propulsion Laboratory, Caltech, PT-JPL) with atmospheric (RH and VPD) and ecophysiological constraints (FPAR and LAI) to downscale potential ET to actual ET. Total ET was the sum of canopy transpiration (ETc), soil evaporation (ETs) and interception evaporation (ETi). Each component was calculated using the Priestley–Taylor equation and the corresponding ecophysiological condition.

MOD16 and PT-JPL methods were applied to estimate global terrestrial LE using the daily MERRA data sets with spatial resolution of 1/2 degree latitude × 2/3 degree longitude, 8 day MODIS FPAR/LAI (MOD15A2) product with 1-km spatial resolution, the 16 day MODIS NDVI (MOD13A2) product with 1-km spatial resolution, the 8 day Global Land Surface Satellite (GLASS) LAI product with 1-km spatial resolution [46], annual Land Cover Type product (MCD12Q1) with 500 m spatial resolution and the Shuttle Radar Topography Mission (SRTM30) Digital Elevation Model (DEM) elevation product. Considering the different spatial resolutions of the MERRA data, spatial interpolation based on the cosine [47] was used to match the spatial resolutions of MERRA and MODIS data.

### 3.2 Ground Measurements

**Fig 2:**
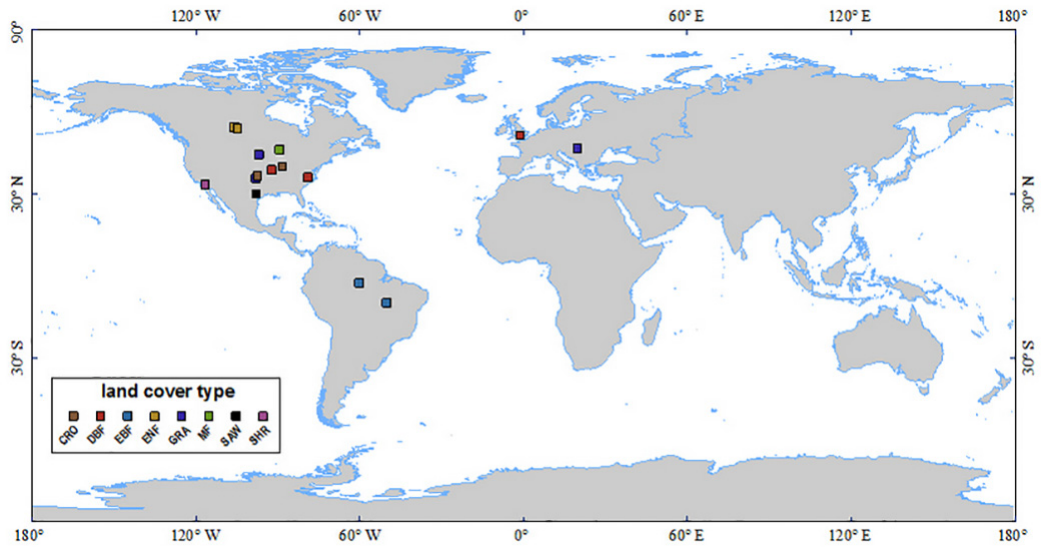
Spatial distribution of the validation FLUXNET sites used in this study. The maps were drawn by the MCD12C1 product for 2005.

The algorithm for EOF integration, MOD16 LE and PT-JPL LE was validated and evaluated using data of 22 EC towers provided by FLUXNET for 2005, as shown in Fig 2. The flux tower sites covered eight major global land-surface biomes: deciduous broadleaf forest (DBF; three sites), deciduous needleleaf forest (DNF; six sites), evergreen broadleaf forest (EBF; two sites), mixed forest (MF; one site), savanna (SAW; one site), shrubland (SHR; one site), cropland (CRO; two sites), and grass and other types (GRA; three sites), as shown in Table 1. The sites were selected according to the following criteria: (a) data being quality controlled; (b) extensive data set with minimal gaps; and (c) availability of all other requireaad input data for simulation using the different models considered for this study. Because of high data availability data from 2005 was selected for this analysis.

**Table 1:** Characteristics of the selected validation data at the FLUXNET sites (S1 Table).

Sites ID	Site name	Latitude	Longitude	IGBP	Available years
US-ARM	ARM Southern Great Plains control site	36.61	-97.49	CRO	2000–2013
US-ARC	ARM Southern Great Plains control site	35.55	-98.04	GRA	2005–2006
US-Bkg	Brookings	44.35	-96.84	GRA	2004–2010
US-Bo1	Bondville	40.01	-88.29	CRO	1996–2010
US-Bo2	Bondville	40	-88.29	CRO	2004–2008
US-Dk2	Duke Forest Hardwoods	35.97	-79.1	DBF	2001–2008
US-FR2	Freeman Ranch- Mesquite Juniper	29.95	-98	SAW	2005–2008
US-MOz	Missouri Ozark Site	38.74	-92.2	DBF	2004–2013
US-SO2	Sky Oaks Old	33.37	-116.62	SHR	
US-SO3	Sky Oaks- Young Stand	33.38	-116.64	SHR	2001–2006
US-SO4	Sky Oaks New	33.38	-116.64	SHR	
US-Syv	Sylvania Wilderness	46.69	-89.35	MF	2001–2008
HU-Bug	Bugacpuszta	46.69	19.6	GRA	2002–2006
UK-PL3	Pang Lambourne (forest)	51.45	-1.27	DBF	2005–2006
BR-Ban	Ecotone Bananal Island	-9.82	-50.16	EBF	2003–2006
BR-Ma2	Manaus—ZF2 K34	-2.61	-60.21	EBF	1999–2006
CA-Obs	Sask.- SSA Old Black Spruce	53.99	-105.19	ENF	1999–2005
CA-Ojp	Sask.- SSA Old Jack Pine	53.92	-104.69	ENF	1999–2005
CA-SF1	Sask.-Fire 1977	54.49	-105.82	ENF	2003–2005
CA-SF2	Sask.-Fire 1989	54.25	-105.88	ENF	2003–2005
CA-SJ2	Sask.-2002 Harvested Jack Pine	53.95	-104.65	ENF	2003–2005
CA-Sj3	Sask.-1975 (Young) Jack Pine	53.88	-104.65	ENF	2004–2005

These data sets included half-hourly or hourly ground-measured incident solar radiation (Rs), relative humidity (RH), air temperature (Ta), diurnal air-temperature range (DT), wind speed (Ws), vapor pressure (e), sensible heat flux (H), surface net radiation [48], ground heat flux (G), and LE. When available, data sets were gap-filled by site principal investigators (PIs), and daily data was aggregated from half-hourly or hourly data without using additional quality control [49–51]. The more detailed information of the validation data were listed in Table 1 and Fig 2. Although the EC technique is regarded as a good method for measuring heat fluxes, the EC based LE values has to be corrected because of the unclosed energy problem (Twine et al. [52]; Wilson et al. [48]). The method developed by Twine et al. [52] was applied to correct LE for all flux towers,

$$LE=\left(R_{n}-G\right)/\left(LE_{ori}+H_{ori}\right)\times LE_{ori}$$ (6)

Where LE is the corrected latent heat flux, H_ori_ and LE_ori_ were the uncorrected sensible heat flux and latent heat flux, respectively.

## 4. Results and Discussion

### 4.1 Comparison of MOD16 and PT-JPL algorithms

**Fig 3:**
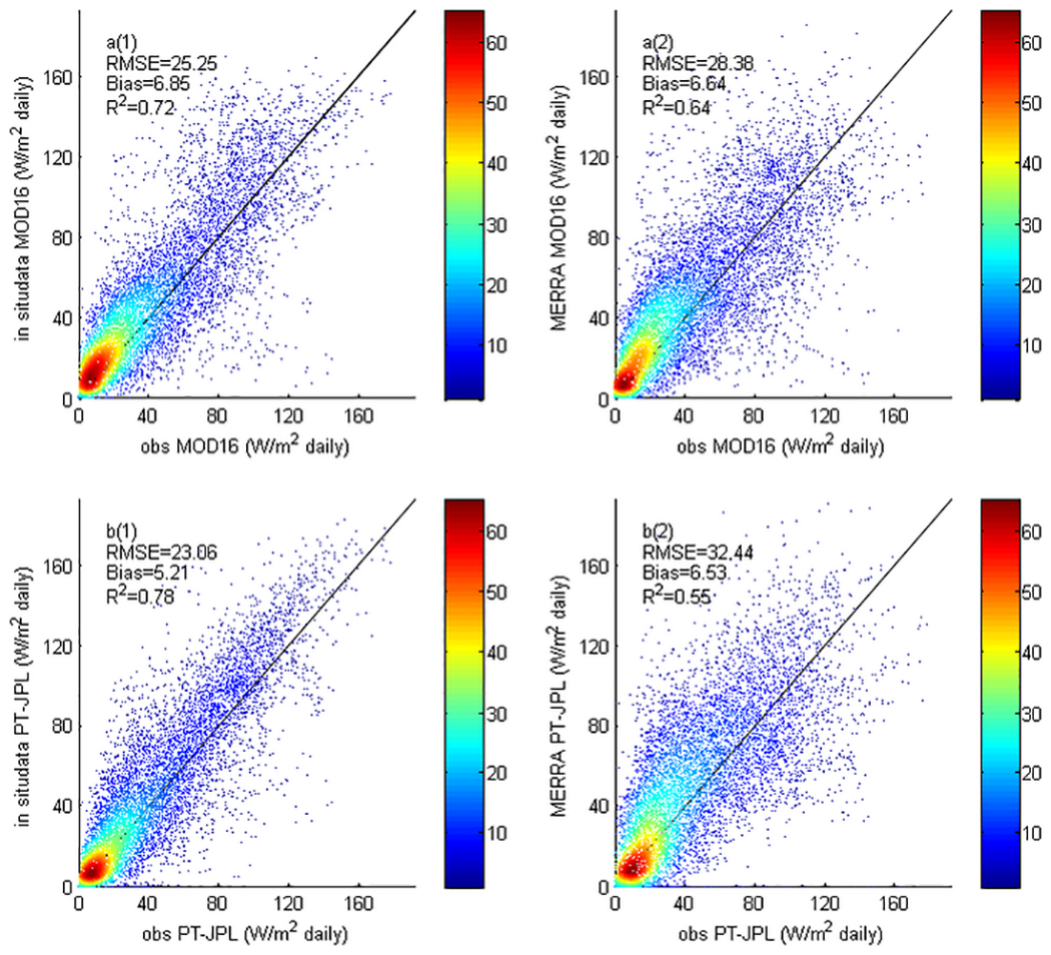
Comparison of MOD16 and PT-JPL with in situ and meteorological forcing data. (a1) MOD16 vs in situ data. (a2) MOD16 vs meteorological forcing data. (b1) PT-JPL vs in situ data. (b2) PT-JPL vs meteorological forcing data.

Daily LE estimates from MOD16 and PT-JPL based on both tower-measured meteorology data and MERRA meteorology data were compared. Furthermore, satellite based LE estimates were also checked against measured LE data for the FLUXNET sites, as shown in Fig 3.

The two models had good performance over these sites. The correlation coefficients, R^2^, of the TP-JPL and MOD16 using in situ meteorology data are higher than 0.6, which corresponds to a good correlation to the measured LE values. Significant differences were found concerning theuncertainties of different algorithms when using in situ and forcing data. A possible reason for the differences might be the scale mismatch issue [53]. The comparison of our validation results with previous studies were in good agreement. Fisher et al. [40] found that PT-JPL had high correlation with ground observations from16 FLUXNET sites. Chen et al. [54] reported that the PT-JPL showed a good performance with R^2^ equal to 0.8. The MOD16 validation at Brazil EC sites conducted by Ruhoff et al. [55] showed that the correlation coefficient between the ground observation and MOD16 estimates for 8-days average was 0.79, RMSE was 0.78 mm day^-1^ and mean bias was 0.54 mm day^-1^. The good performances of MOD16 and PT-JPL might be attributed to good physical basis of these two models. However, previous studies also showed that MOD16 had reduced performance when compared to ground observations. Chen et al. [4] and Ershadi et al. [20] reported reduced performance of MOD16 compared with Chinaflux EC sites and American EC sites. MOD16 validation conducted by Ramoelo et al. [56] suggested that disagreement of MOD16 and flux tower-based ET could be attributed to the parameterization of Penman-Monteith model. Therefore, the parameterization of Penman-Monteith model at different sites or climatic zones might cause the different performances of MOD16.

Small discrepancies in LE were produced by MOD16 and PT-JPL (Fig 3). Both MOD16 and PT-JPL showed positive bias compared with ground measurements. However, as published by Behrangi et al. [57], the MOD16 slightly underestimates LE as compared to PT-JPL. Moreover, when compared with EC towers in Asia, MOD16 had a negative bias (-17.00 mm 8-day^-1^) especially for the cropland sites [58]. This might be due to the different location of the ground observations, i.e. most of EC sites in this study were collected at high latitudes (Fig 1) and in high latitudes, temperature has great impact on LE estimations [59].

**Fig 4:**
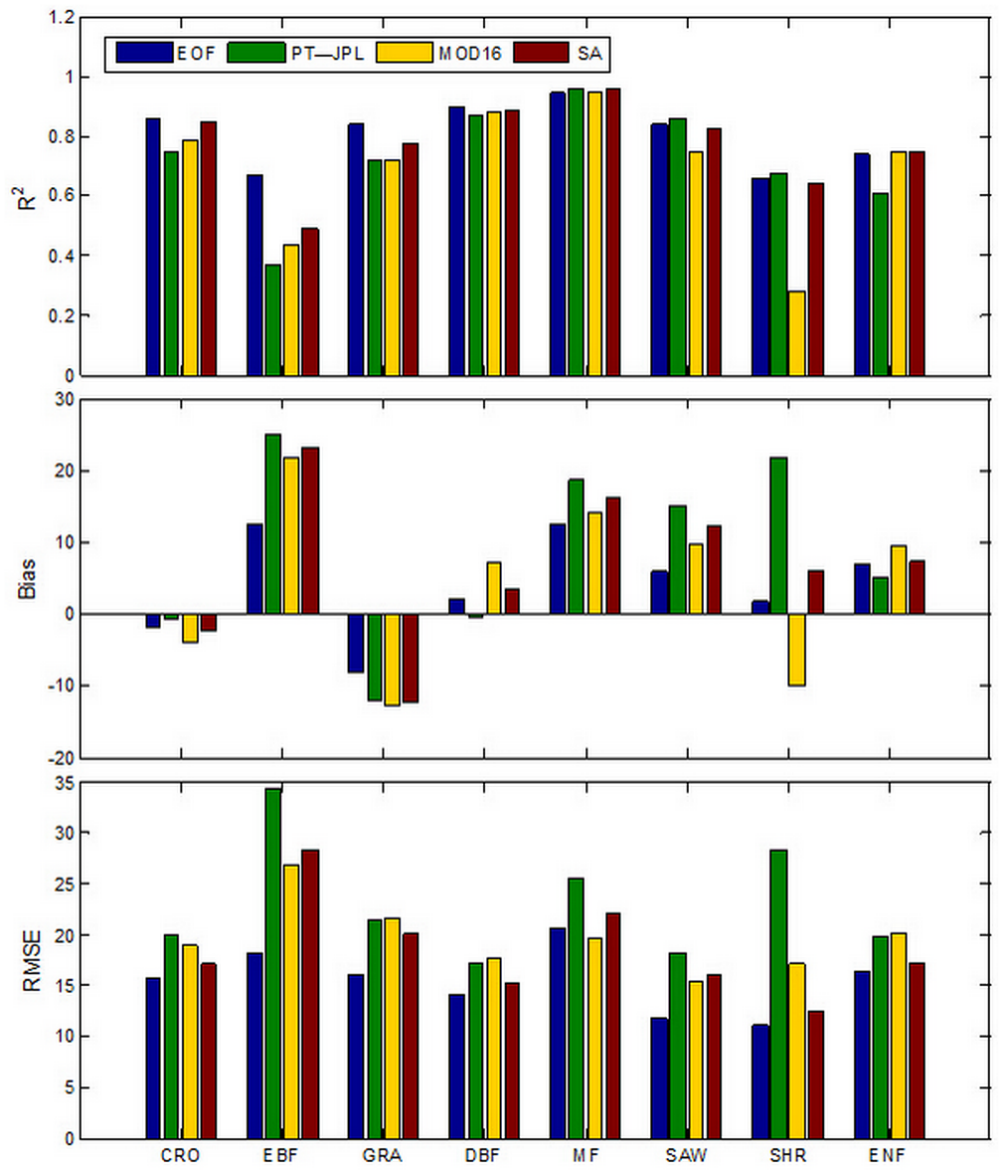
Direct validation results of EOF-integrated LE for FLUXNET sites at eight biomes: cropland (CRO), evergreen broadleaf forest (EBF), grassland (GRA), deciduous broadleaf forest (DBF), mixed forest (MF), savanna (SAW), shrubland (SHR) and evergreen needleleaf forest (ENF).

We found that vegetation type has a great influence on the performance of MOD16 and PT-JPL (Fig 4). PT-JPL showed the higher R^2^ (0.96) for MF sites, while MOD16 had the higher R^2^ (0.75) for the ENF sites. PT-JPL generally had lower bias than MOD16 for CRO, GRA, DBF and ENF, whereas the MOD16 showed a lower bias than PT-JPL for EBF, MF, SAW and SHR. Both algorithms showed negative bias for CRO and GRA sites. The underestimation of MOD16 for cropland site was also reported in the previous study [58]. Yao et al. [28] also found that MOD16 and PT-JPL underestimates LE in the case of cropland and grassland sites. Negative biases in simulated LE by these two algorithms might be attributed to the uncertainty in soil moisture estimation.

### 4.2 Performance of EOF and SA fusion algorithms

**Fig 5:**
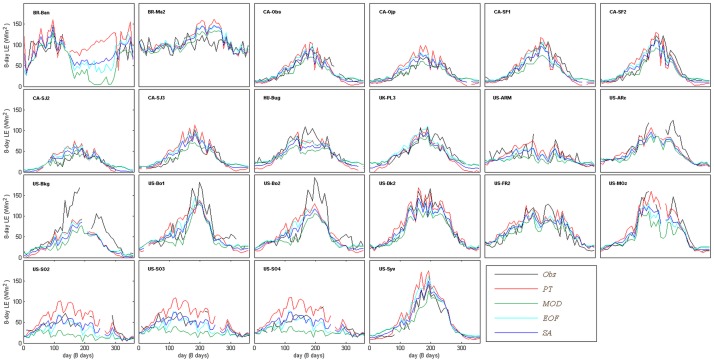
Validation of the 8-day mean of predicted and observed LE at all sites in 2005.

The MOD16, PT-JPL, SA and EOF algorithms exhibited substantial differences when comparing the modelled LE to the LE observed at the 22 EC flux tower sites, as shown in Fig 5. However, MOD16, PT-JPL, SA and EOF algorithms successfully predicted the magnitudes and seasonal variations of the observed LE at the validation sites. Compared with MOD16 and PT-JPL, the fusion algorithms (SA and EOF) showed closer correlation with observed LE, LE predicted by EOF being the best estimate for most sites. Generally, previous studies also showed that fusion methods could produce more accurate LE estimates than the individual LE algorithm. Ershadi et al. [20] found that the ensemble mean of the individual LE models produced the best estimates of LE, with the mean value of the Nash–Sutcliffe efficiency of 0.61 and the root mean squared difference of 64 W/m^2^. Yao et al. [28] introduced a Bayesian model averaging (BMA) method by merging five process-based LE models. This BMA method showed improved performance compared with individual LE models from 240 FLUXNET EC sites R^2^ being equal to 0.8, bias equal to 3.5 W/m^2^ and RMSE equal to 32.8 W/m^2^.

For validation the 22 sites were categorized according to land cover type, as shown in Fig 4. Both EOF and SA showed higher R^2^ than MOD16 and PT-JPL for CRO, EBF, GRA and DBF ranging from 0.49 to 0.86. In the case of CRO, EBF and GRA sites, LE estimate by EOF showed the highest correlation ranging from 0.67 to 0.86. In terms of RMSE EOF produced the lowest values ranging from 11.07 to 18.10 W/m^2^ for all biomes. Except for DBF and ENF sites bias was the lowest for EOF. SA had a relative good performance with bias ranging from -12.37 to 23.40 W/m^2^ and RMSE ranging from 15.39 to 28.37 W/m^2^ over CRO, GRA and DBF sites. However, SA showed limited improvement for EBF, GRA and MF sites compared with MOD16 and PT-JPL. However there is a variety of individual LE models with different algorithm structures and parameterization, none of them is capable of providing a best LE estimate for all biomes. Hence, the reduced performance of SA might be attributed to the simple constant weights of different LE models [28]. Because EOF took both spatial and temporal information into consideration when reconstructing LE and maintained the main spatial pattern of individual LE model [35], compared to SA, EOF provided improved performance over most vegetation types. The previous study [37] showed that fusion methods based on EOF had substantially improved the accuracy of LAI with R^2^ increasing from 0.75 to 0.81 and RMSE decreasing from 1.04 to 0.71. The simple structure of EOF fusion might partly explain this improvement. The major advantage of EOF was that it avoided using the measured LE values which were used by many of the fusion methods [28]. Therefore the sensitivity of this parameterization to errors in the input data was substantially lessened [60]. Another advantage was that it was easy to implement and did not require the use of a precalculated covariance model and estimation error matrix [37].

**Fig 6:**
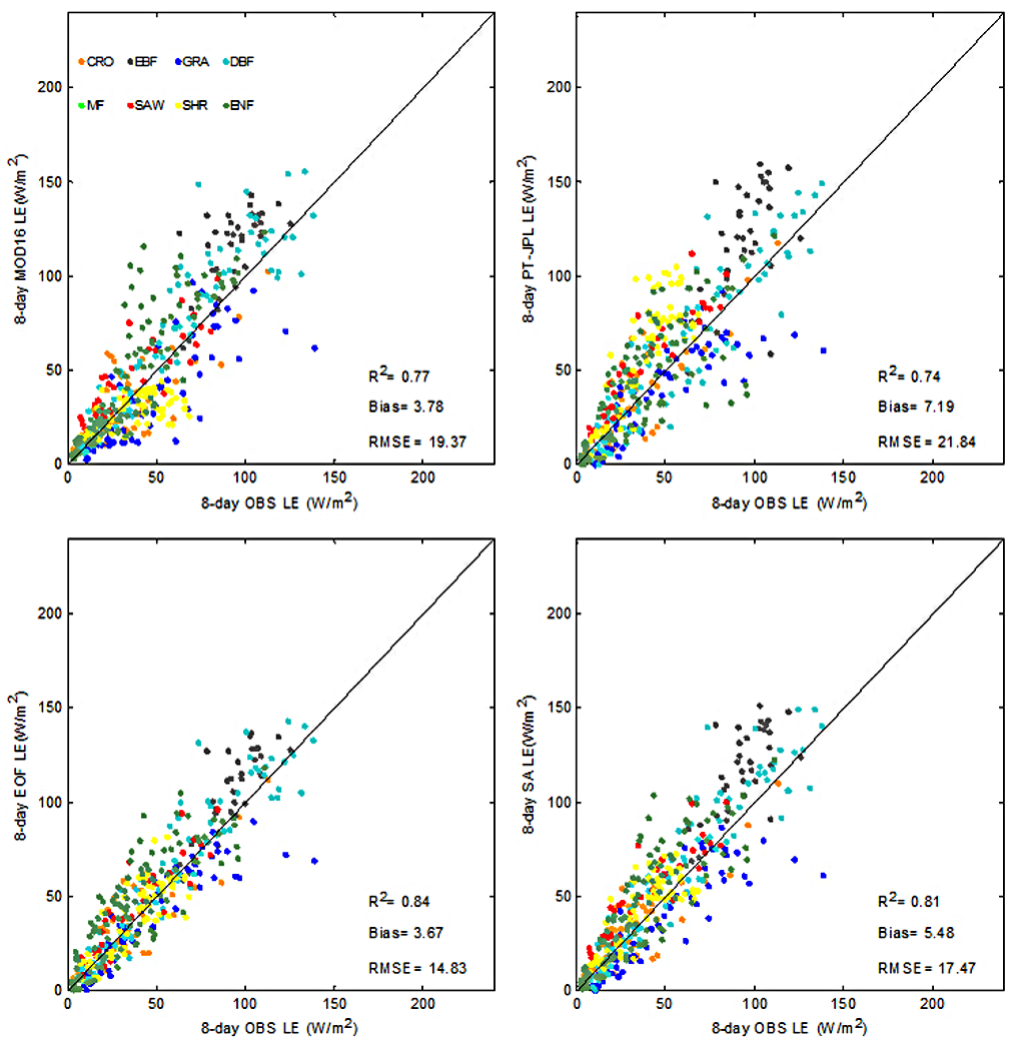
Validation of EOF, MOD16 and PT-JPL LE methods across different land use types. 8 day average LE prediction is compared to ground measurements. The solid line is the 1:1 line.

Considering all vegetation types (all validation sites), MOD16, PT-JPL, EOF and SA explained 77%, 74%, 84% and 81% of the variation of the 8 day average LE estimates, respectively (Fig 6), with MOD16 slightly overestimating and PT-JPL overestimating LE. All methods showed positive bias ranging from 3.67 W/m^2^ to 7.19 W/m^2^ and RMSE was in the range of 14.83 W/m^2^ and 21.84 W/m^2^. The proposed EOF method showed then lowest bias and RMSE and the highest R^2^ (0.84).

**Fig 7:**
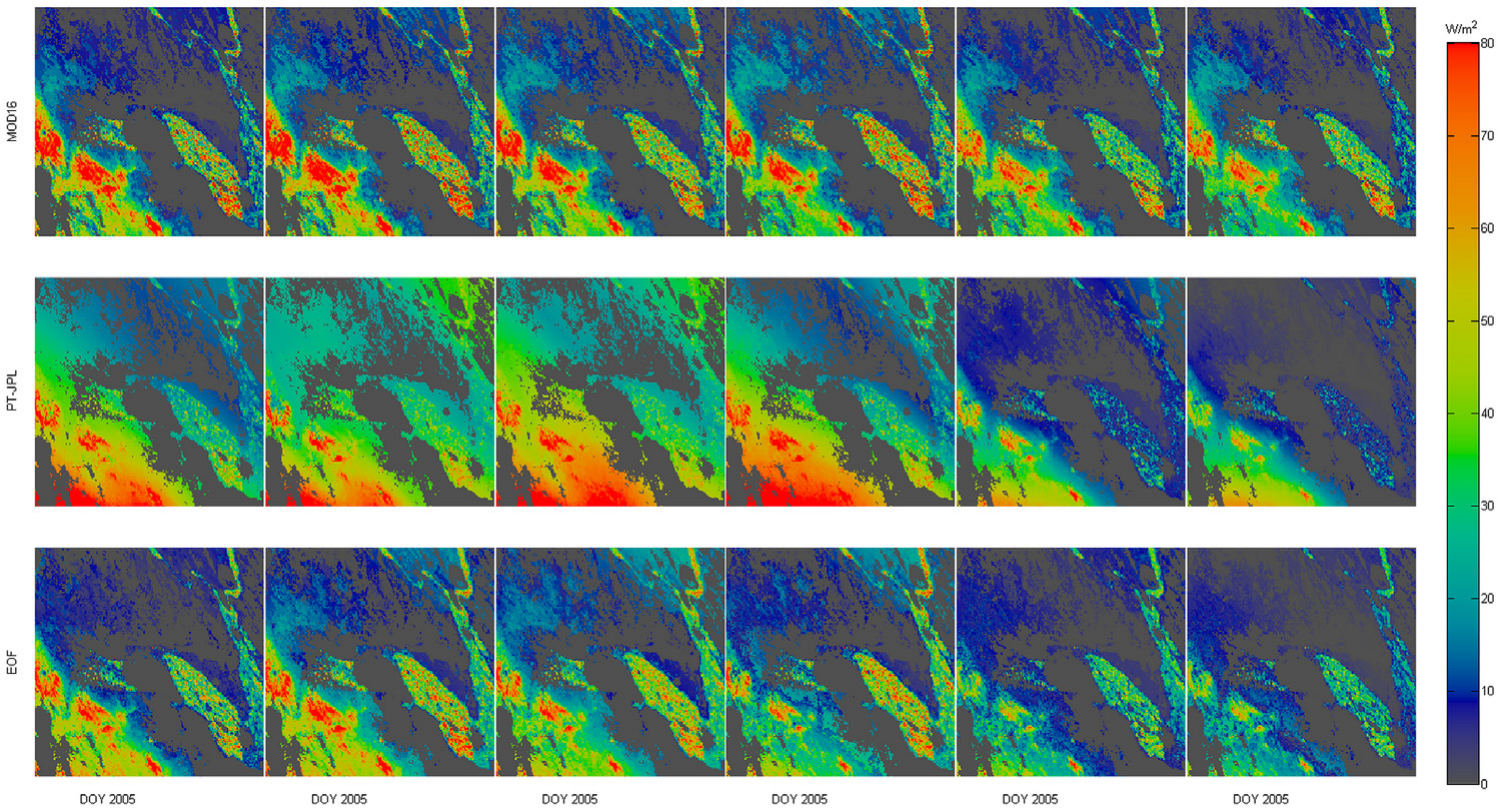
EOF maintained major pattern of PT-JPL and removed the extreme values as compared with the SA, MOD16 and PT-JPL methods during the periods from February 10, 2005, to March 22, 2005, at the US-SO2 AmeriFlux Site. The color bar is an 8-day composite LE. Dark gray color means no data (0 value).

To compare spatial patterns of LE in the case of the four algorithms, we randomly selected images around US-SO2 site (Fig 7). Similar tendencies of LE predicted by the four algorithms were found, LE was decreasing regularly with time. As expected, for a given day the largest difference was between LE predicted by SA and EOF. Compared to SA, EOF showed relatively low LE in upper-left corner of the images, which was more consistent with the LE predicted by MOD16. EOF and SA both showed high LE in bottom left corner of the images. However, the SA showed distinct average results of MOD16 and PT-JPL.

### 4.3 Limitations of the proposed EOF algorithm

The proposed EOF algorithm requires fewer input parameters than geostatistical approaches, e.g. a precalculated covariance model. However, EOF does have some limitations, and the computation cost is very high due to matrix calculations and iterations. Uncertainties that are limiting the use of the EOF model are the following:

1. The bias for MERRA [61] might lead to substantial bias for the two individual LE algorithms and hence to the EOF ensembles. Study showed that MERRA surface solar radiation, which was used as an input of LE models, had an average bias error of +20.2 W/m^2^ on monthly and annual scales from American FLUXNET sites [62], resulting in an overestimation of LE. Zib et al. [63] also reported an annual mean bias of 3.9 W/m^2^ at two Baseline Surface Radiation Network (BSRN) sites for MERRA surface solar radiation; Wang and Zeng [64] found an overestimation of up to 40 W/m^2^ for MERRA surface solar radiation.
2. Scale mismatch between coarse resolution of input data and the field measurements footprint may results in substantial bias in EOF fusion producing. Wolde et al. [65] analyzed the different pixel resolution of remote sensing inputs, and showed that variation in ET flux between corn and soybean field could not be effectively distinguished when the input was of the order of 1000 m [66] found that coarse NCEP/NCAR reanalysis meteorology (NNR) data can introduce bias to match the local tower footprint in some regions.
3. By integrating the different LE algorithms, bias might be introduced during the EOF fusion process. The reason it that, when applying the EOF fusion method, EOF reconstruction scheme [42] does not distinguish between good or degraded quality pixel values. Consequently, bias is introduced, since all pixels in the image are included in the reconstruction during the spatio-temporal fusion process.

## 5. Conclusions

We proposed a data merging method based on EOF analysis and applied this method to integrate two satellite-derived LE products (MOD16 and PT-JPL). We also compared the proposed EOF method with simple SA fusion method. Ground-measured LE data in 2005 from 22 EC sites, incorporating eight major terrestrial biomes (CRO, DBF, EBF, ENF, GRA, MF, SAW and SHR), were used for validation, and demonstrated that the proposed method was suitable for terrestrial LE mapping.

MOD16 and observed data correlated well for EBF, MF, SAW and SHR biomes, producing higher R^2^, although somewhat larger RMSE and high bias. For CRO, GRA, DBF and EOF sites, PT-JPL produced lower bias with lower RMSE. Although SA fusion method provided acceptable results compared with MOD16 and PT-JPL, the proposed EOF algorithm showed notable improvement by combining the advantages of MOD16 and PT-JPL and had a relatively low bias and RMSE with high R^2^ for all biomes. EOF integrated images were superior to LE maps generated by the PT-JPL and MOD16 algorithms.

## Supporting Information

S1 File The EOF program and the validation result. the program were contained in the S1 File.rar. The EOF program were derived in IDL(.pro). the validation results were in the Excel tables.
(RAR)

S1 Table Validation sites description. The Characteristics of the the FLUXNET sites. Data was in the S1 Table.xlsx.
(XLSX)
